# The radiological characteristics of advanced orbital squamous cell carcinoma in patients who underwent orbital exenteration in a tertiary centre, South Africa

**DOI:** 10.4102/jcmsa.v4i1.404

**Published:** 2026-10-02

**Authors:** Nomcebo O. Mkhatshwa, Mokokomadi A. Makgotloe, Thandi Buthelezi, Craig D. Anderson

**Affiliations:** 1 Division of Ophthalmology, Department of Neurosciences, Faculty of Health Sciences, University of the Witwatersrand, Johannesburg, South Africa; 2 Department of Diagnostic Radiology, School of Clinical Medicine, Faculty of Health Sciences, University of the Witwatersrand, Johannesburg, South Africa

**Keywords:** advanced, squamous cell carcinoma, orbit, exenterarion, St John Eye Hospital

## Abstract

**Background:**

Orbital squamous cell carcinoma (SCC) is a rare entity. It is a locally invasive malignancy which spreads to orbital structures, and if left untreated, can spread intracranially. The definitive treatment for SCC which is confined to the orbit is orbital exenteration. The aim of this study was to describe the radiological features of orbital SCC in patients who underwent orbital exenteration at a tertiary eye hospital in South Africa.

**Methods:**

A retrospective cross-sectional study of 51 patients with histologically confirmed advanced orbital SCC, who underwent orbital exenteration between 01 January 2018 and 31 December 2023, analysing the radiological characteristics on computed tomography scans taken prior to exenteration.

**Results:**

The mean age was 46.7 years (s.d. 13.6), and 63% patients were male. The population was mostly African (88%), with a significant proportion (82%) testing positive for Human Immunodeficiency Virus (HIV). Majority of tumours were confined to the orbit (57%) with even laterality. Globe involvement was extensive (90%), with lacrimal gland involvement in 41% of patients. Muscle involvement was also observed with the inferior rectus muscle most affected (58%), followed by the medial rectus muscle (48%). Patients with disease confined to the orbit were less likely to have lymph node involvement (*p* = 0.001) and distant metastatic spread (*p* = 0.001). In contrast, optic nerve spread groups showed more aggressive features, with significant differences observed in lymph node involvement (*p* = 0.001), bone erosion (*p* = 0.005) and overall sinus involvement (*p* = 0.001).

**Conclusion:**

Squamous cell carcinoma requiring exenteration is varied in its presentation.

**Contribution:**

Majority of patients were HIV positive. Optic nerve spread of the tumour had significantly more aggressive features.

## Introduction

Primary orbital squamous cell carcinoma (SCC) is a rare entity in the developed world with only 15 cases reported in literature worldwide.^1^ Advanced orbital SCC is usually found to arise secondarily from another source. The presumed origin of advanced orbital SCC in such cases is local invasion with perineural spread, arising from adjacent structures such as periorbital skin, ocular surface such as the conjunctiva, paranasal sinuses and ocular adnexa.^1^ The outcomes of advanced orbital SCC can be devastating, being both sight and life threatening with no universally accepted treatment once the tumour spreads extensively. The choices include surgical excision, radiation and chemotherapy among others.^1,2,3^ Over the last four decades, the global incidence has risen attributed to the increased exposure to carcinogens and the increasing life expectancy in people living with Human Immunodeficiency Virus (HIV)/Acquired Immunodeficiency Syndrome (AIDS) and associated human papilloma virus.^3,4^

With the growing incidence of HIV and AIDS and associated Human papilloma virus (HPV) infections, particularly subtypes 16 and 18, SCC is becoming more prevalent with estimates of incidence rates as high as 3.4 per 100 000, for conjunctival SCC from sub-Saharan Africa.^3,5,6^ Other risk factors include chronic ultraviolet radiation exposure from sunlight, smoking and fair skin.^7^

Orbital SCC is a locally aggressive tumour and can infiltrate surrounding structures. The paranasal sinuses can be affected once there is extensive orbital involvement where the ethmoidal sinus followed by the maxillary sinus are the commonest sinuses involved.^8,9^ Once there is perineural invasion then the risk of distant spread and intracranial involvement increases.^10,11^

Radiological characteristics are reported to be variable. Contrast-enhanced computed tomography (CT) and magnetic resonance imaging (MRI) scans provide valuable information about the tumour as they assess regional nodal status, paranasal sinuses, orbit, brain and chest which assist in planning management.^12^ The Royal College of Radiologists has protocols for CT and MRI scans for patients with orbital SCC. Computed tomography protocol includes spiral CT scan with intravenous contrast injection, and 3 mm thickness slices whilst MRI scan protocol includes pre- and post-gadolinium T1 weighted (T1W) and post gadolinium T1 fat suppressed (STIR – short tau inversion recovery images) at a thickness less than 4 mm and includes axial and coronal views.^13,14^

Management ranges from primary orbital radiation and observation, orbital exenteration, adjuvant chemotherapy and radiation.^15^ Once the malignancy is advanced, patients usually require exenteration, especially when musculature is involved.^4,14,15^

Orbital exenteration is a highly disfiguring operation and often the final measure in managing advancing disease involving the orbit. Wang et al.^16^ reported that a 20-year study found malignancies in the orbit and 90% of the patients underwent orbital exenteration. There is a relative paucity of research on orbital exenteration especially within sub-Saharan Africa. A study by Aryasit et al.^17^found that the most common indication for orbital exenteration in their study patients was SCC, 34% of which were involving the eyelid and conjunctiva. We therefore sought to describe the radiological features of orbital SCC in patients who underwent orbital exenteration at a tertiary ophthalmic referral hospital.

## Research methods and design

This was a retrospective cross-sectional study of CT scans of 51 adult patients, above the age of 18 years, who presented with histologically confirmed advanced SCC of the orbit and were all treated with an exenteration procedure at St John Eye Hospital in Soweto, South Africa (a tertiary Ophthalmic referral hospital). Patients had to be above 18 years of age at the time of treatment, treated between the 01 January 2018 and 31 December 2023. All the patients had CT scans performed as part of their pre-operative assessment. This study primarily describes features of these tumours as viewed on the patients’ CT scans.

### Data collection

Data were collected from the St John Eye Hospital theatre record books and included patients who were surgically operated from 01 January 2018 to 31 December 2023. Patient’s CT scan studies and reports were obtained to assess the stage at the time of pre-operative assessment.

Study data were collected anonymously and managed using the Research Electronic Data Capture (REDCap) data management system, hosted at the University of the Witwatersrand.^18^ Each participant was allocated a randomly generated Redcap participant number to conceal their identity.

Statacorp.2023. Statistical software: Stata 18, StataCorp LLC, College Station, Texas, United States, was used for statistical analysis. The summary used mean (s.d. = standard deviation) /median (interquartile range) for continuous variables. Frequencies and percentages were used for categorical variables. For associations, Chi square or Fisher’s exact test was used in categorical variables and analysis of variance (ANOVA)/Kruskal Wallis was used for continuous and categorical variables.

The obtained data were analysed according to the following broad categories: patient demographics and HIV status, laterality of the tumour, histological differentiation of the tumour and keratinisation, radiological characteristics of the tumour and surrounding structures involved, bone erosion in the orbit and muscle involvement by the tumour.

This was a retrospective study reviewing medical records and radiological images. The study was conducted in compliance with the Declaration of Helsinki revised in 2013 and with anonymisation of patient data to maintain confidentiality was implemented. Ethics approval to conduct the study was provided by the Wits Health Research and Ethics Committee (HREC clearance number: M250281), strict compliance to the Protection of Personal Information Act (POPIA) (https://popia.co.za) laws and regulations were followed.

### Ethical considerations

Ethical clearance to conduct this study was obtained from the University of the Witwatersrand Human Research Ethics Committee (No: M250281).

## Results and Discussion

Our study included a total number of 51 adult patients above the age of 18 years. In sub-Saharan Africa, SCC is common in younger patients with chronic acquired immunosuppression and is associated with worse outcomes.^19^ The mean age in our study population was 46.7 (s.d. 13.6) (Table 1). Elderly patients (mean age = 60.6 years) had significant lymph node involvement compared to younger patients (mean age = 45.2) who did not have lymph node involvement (*p*-value 0.015), therefore the average age of those with lymph node involvement is greater than those without (Table 2). Although lymph node involvement is a well-established prognostic factor in many head and neck tumours, ophthalmic literature has little information about lymph node involvement in orbital SCC.^20^ This is attributed to the absence of lymph nodes in the orbit and therefore the tumour could have originated outside of the orbit, conjunctiva or skin.

**TABLE 1:** Patients’ demographics, clinical and radiological variables.

Characteristic	*n*	%	Mean
**Age**	-	-	46.7
**Gender**			
Female	19	37	-
Male	32	63	-
**Race**			
African people	45	88	-
Indian and/or Asian people	1	2	-
Caucasian people	5	10	-
**HIV status**			
Negative	4	8	-
Positive	42	82	-
Unknown	5	10	-
**Radiology findings**			
Confined to orbit	29	57	-
Optic nerve spread	15	29	-
Other	4	8	-
Unknown	3	6	-
**Laterality**			
Left	25	49	-
Right	26	51	-
**Globe involvement**			
No	5	10	-
Yes	46	90	-
**Lacrimal gland involvement**			
No	30	59	-
Yes	21	41	-
**Lymph node involvement**			
No	46	90	-
Yes	5	10	-
**Bone erosion**			
No	40	78	-
Yes	11	22	-
**Intracranial spread**			
No	46	90	-
Yes	5	10	-
**Distant metastasis**			
No	44	86	-
Yes	7	14	-
**Frontal sinus**			
No	48	94	-
Yes	3	6	-
**Ethmoid sinus**			
No	41	80	-
Yes	10	20	-
**Sphenoid sinus**			
No	50	98	-
Yes	1	2	-
**Maxillary sinus**			
No	47	92	-
Yes	4	8	-
**Total sinuses involved**			
0	39	76	-
1	6	12	-
2	2	4	-
3	1	2	-
4	1	2	-

HIV, human immunodeficiency virus; s.d., standard deviation.

**TABLE 2:** Age and main radiological findings.

Characteristic	Mean age	s.d.	*p*-value
**Lymph node involvement**	-	-	0.0150
No	45.2	12.5	-
Yes	60.6	16.9	-
**Bone erosion**	-	-	0.0847
No	45.0	13.2	-
Yes	53.0	13.9	-
**Intracranial spread**	-	-	0.0504
No	45.5	12.6	-
Yes	58.0	18.7	-
**Distant metastasis**	-	-	0.1545
No	45.6	12.4	-
Yes	53.6	19.2	-
**Ethmoid sinus**	-	-	0.4318
No	45.9	12.6	-
Yes	49.8	17.5	-

s.d., standard deviation.

Our study did not demonstrate any statistically significant correlation between age and likelihood of bone erosion (*p* = 0.085) and distant metastasis (*p* = 0.155) (Table 2). There are limited studies that reported on the relationship between age and radiological characteristics or tumour behaviour of orbital SCC, with some case reports on patients with orbital SCC indicating a preponderance of patients above the age of 60 years.^21,22,23^

A large proportion of our study population were African (88%), followed by Caucasian (10%) (Table 1). A study by He et al. in Shanghai, China found that the total incidence of non-cutaneous SCC of the orbital region from 1975 to 2019 was 0.68 per 1 000 000 persons with a steady increase over the years (Annual Percentage Change [APC] 0.69, 95% confidence interval [CI] 0.184–1.192; *p* < 0.05). Further incidence of non-cutaneous SCC of the orbital region was reported to be 0.72 (APC 0.85; 95% CI; 0.297–1.409; *p* < 0.05%) in Caucasians which was significantly higher than other races.^11^ Our hospital’s patients’ race distribution is almost entirely African. Therefore, race distribution in our study is not representative of the general population.

He et al.,^11^ also reported the incidence of non-cutaneous orbital SCC to be higher in men (1.16) than women (0.32). A total of 63% of our study patients were male and 37% were female (Table 1). An extensive review of previously reported cases in the literature revealed substantial heterogeneity in demographic, clinical and therapeutic profiles. Nath et al.^21^ reported no gender predilection in orbital SCC.

Analysis of gender preponderance in our study illustrates that radiological characteristics of advanced orbital SCC were comparable across both genders.

Other parameters were similar between both genders such as HIV status, laterality, globe involvement, lacrimal gland and lymph node involvement, intracranial and metastatic spread demonstrating no statistical significance (*p* = 1.000). Our reports did not specify or distinguish the orbital part from the palpebral part of the lacrimal gland in terms of involvement.

Tumour differentiation highlights the aggressive nature of this tumour and tendency to spread quite rapidly beyond the primary site. The majority of our study patients had moderately differentiated at 52.9% followed by those having poorly differentiated tumours at 37.2% (Figure 1). This was similar to what a 2025 retrospective case series including an Australian and New Zealand Cohort of 10 patients with orbital SCC, found that the tumours ranged from moderately to poorly differentiated SCC. The patients with poorly differentiated tumours had the worst outcome.^24^

**FIGURE 1:**
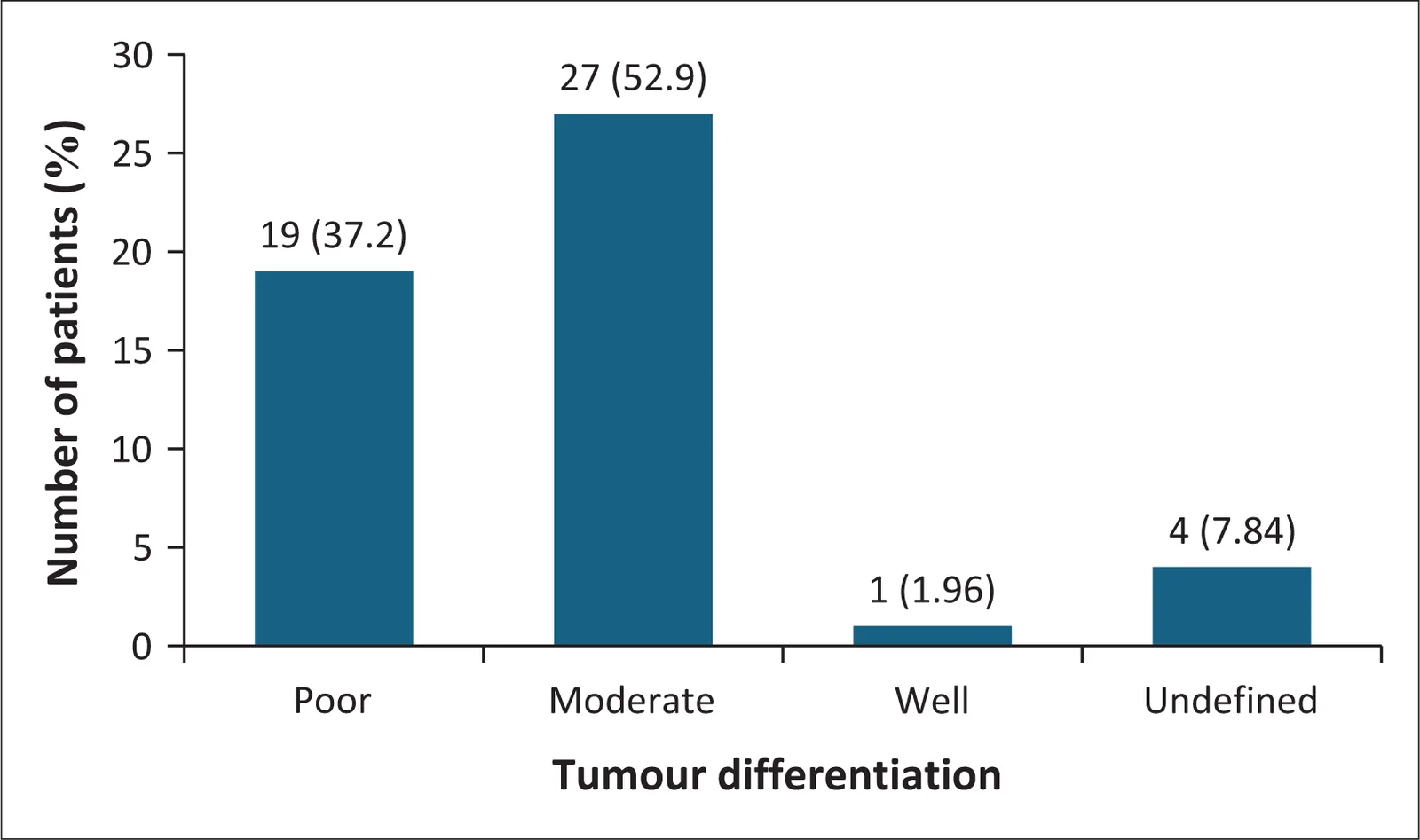
Vertical bar graph showing degree of histological differentiation.

Delayed patient referral rather than intrinsic tumour’s biological features could be a contributing factor. An epidemiological study carried out by Van Wyk et al.^25^ in South Africa in 2025 reported that one of the reasons of delayed presentation in some patients was that they initially ignored the tumour until it grew large prior to seeking healthcare assistance.

The majority of tumours in our study population (56.9%) were confined to the orbit. This was followed by 37.3% of the patients demonstrating optic nerve spread (Figure 2). Orbital SCC has variable radiological characteristics as evidenced by case reports published in the literature. A case report by Alasdair et al.^22^ reported that their patient had a lesion in the left orbit extending to the cavernous sinus without any paranasal sinus pathology. The Australian and New Zealand cohort found that lesions in their patients’ scans were extraconal, infraorbital margin, inferior, roof, orbital apex, floor and lateral orbit.^24^ Korchnoy et al.^23^ found in their case report that their patient had a tumour involving the right sphenoid bone invading the lateral extraconal orbit. All these findings reiterate the varied presentation of this malignancy, which was demonstrable in our study population.

**FIGURE 2:**
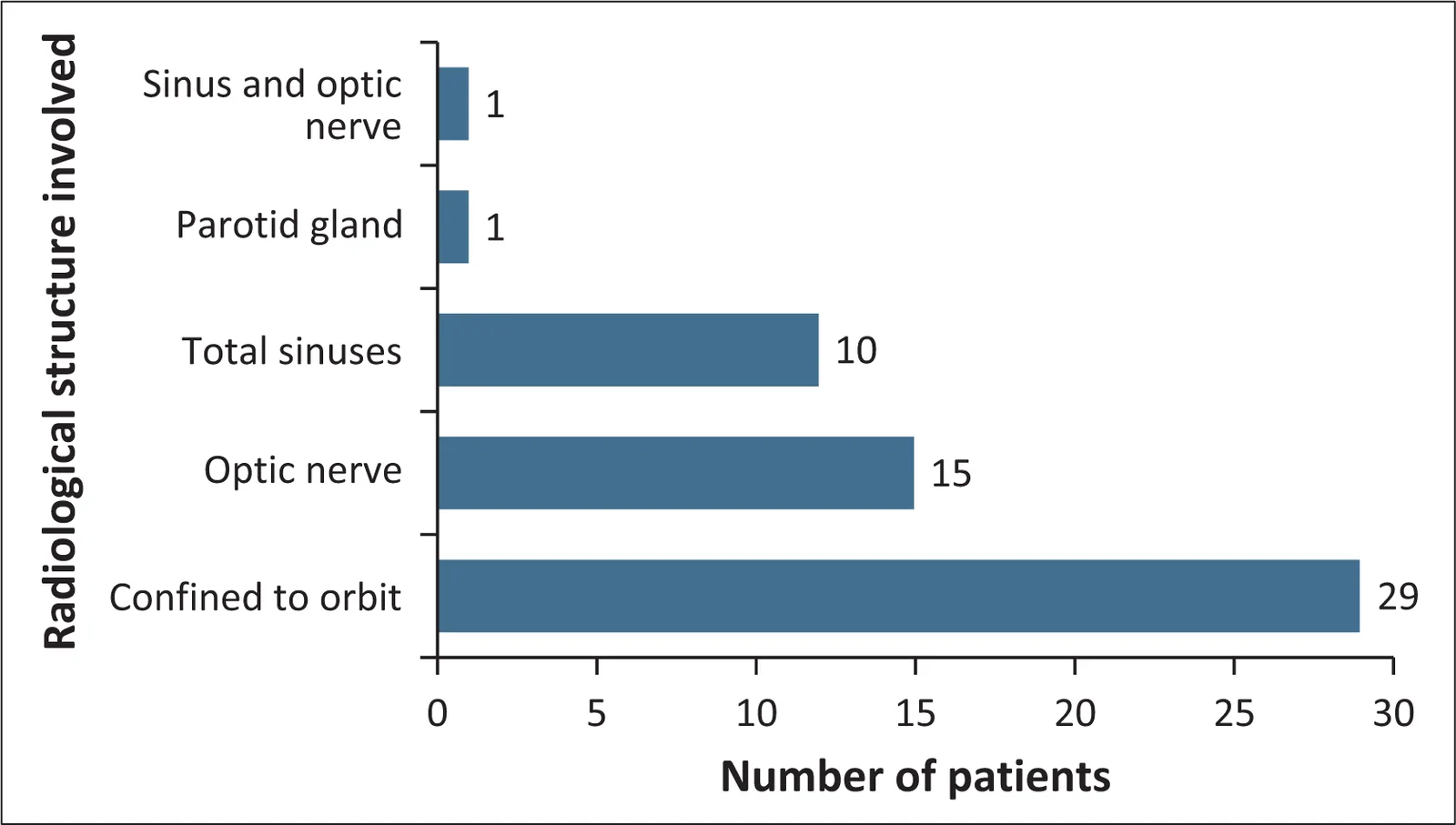
Horizontal bar graph showing distribution (in numbers) of radiological findings among the patients.

Patients who had optic nerve spread demonstrated aggressive disease with statistically significant involvement of lymph nodes (*p* = 0.001), bone erosion (*p* = 0.005), intracranial spread (*p* = 0.006), distant metastasis (*p* = 0.001) and sinus spread (*p* = 0.0001). We found that the larger the tumour dimension, the higher the morbidity associated with tumour spread beyond the orbit (*p* = 0.0092) (Table 3). Although we found this in our study, further studies need to be conducted looking at tumour dimension and degree of associated morbidity and tendency towards tumour spread.

**TABLE 3:** Radiological, clinical and histological findings according to key spread patterns.

Characteristic	Confined to orbit				Optic nerve				Other				*p*-value
	*n*	%	Median	IQR	*n*	%	Median	IQR	*n*	%	Median	IQR	
**Age (Years)**	48.7	12.3	-	-	42.5	14.1	-	-	50.3	19.9	-	-	0.4450
**Gender**	-	-	-	-	-	-	-	-	-	-	-	-	0.4980
Female	9.0	31.0	-	-	8.0	53.0	-	-	1.0	25.0	-	-	-
Male	20.0	69.0	-	-	7.0	47.0	-	-	3.0	75.0	-	-	-
**HIV status**	-	-	-	-	-	-	-	-	-	-	-	-	0.3530
Negative	3.0	10.0	-	-	0.0	-	-	-	1.0	25.0	-	-	-
Positive	24.0	83.0	-	-	13.0	87.0	-	-	3.0	75.0	-	-	-
Unknown	2.0	7.0	-	-	2.0	13.0	-	-	0.0	-	-	-	-
**Laterality**	-	-	-	-	-	-	-	-	-	-	-	-	0.6030
Left	14.0	48.0	-	-	6.0	40.0	-	-	3.0	75.0	-	-	-
Right	15.0	52.0	-	-	9.0	60.0	-	-	1.0	25.0	-	-	-
**Globe involvement**	-	-	-	-	-	-	-	-	-	-	-	-	0.3160
No	5.0	17.0	-	-	0.0	-	-	-	0.0	-	-	-	-
Yes	24.0	83.0	-	-	15.0	100.0	-	-	4.0	100.0	-	-	-
**Lacrimal gland involvement**	-	-	-	-	-	-	-	-	-	-	-	-	0.0650
No	17.0	59.0	-	-	11.0	73.0	-	-	0.0	-	-	-	-
Yes	12.0	41.0	-	-	4.0	27.0	-	-	4.0	100.0	-	-	-
**Lymph node involvement**	-	-	-	-	-	-	-	-	-	-	-	-	0.0010
No	29.0	100.0	-	-	13.0	87.0	-	-	1.0	25.0	-	-	-
Yes	0.0	-	-	-	2.0	13.0	-	-	3.0	75.0	-	-	-
**Bone erosion**	-	-	-	-	-	-	-	-	-	-	-	-	0.0050
No	27.0	93.0	-	-	8.0	53.0	-	-	2.0	50.0	-	-	-
Yes	2.0	7.0	-	-	7.0	47.0	-	-	2.0	50.0	-	-	-
**Intracranial spread**	-	-	-	-	-	-	-	-	-	-	-	-	0.0060
No	29.0	100.0	-	-	12.0	80.0	-	-	2.0	50.0	-	-	-
Yes	0.0	-	-	-	3.0	20.0	-	-	2.0	50.0	-	-	-
**Distant metastasis**	-	-	-	-	-	-	-	-	-	-	-	-	0.0010
No	29.0	100.0	-	-	10.0	67.0	-	-	2.0	50.0	-	-	-
Yes	0.0	-	-	-	5.0	33.0	-	-	2.0	50.0	-	-	-
**Frontal sinus**	-	-	-	-	-	-	-	-	-	-	-	-	0.0120
No	29.0	100.0	-	-	14.0	93.0	-	-	2.0	50.0	-	-	-
Yes	0.0	-	-	-	1.0	7.0	-	-	2.0	50.0	-	-	-
**Ethmoid sinus**	-	-	-	-	-	-	-	-	-	-	-	-	0.0010
No	28.0	97.0	-	-	8.0	53.0	-	-	2.0	50.0	-	-	-
Yes	0.0	-	-	-	7.0	47.0	-	-	2.0	50.0	-	-	-
**Sphenoid sinus**	-	-	-	-	-	-	-	-	-	-	-	-	0.1370
No	29.0	100.0	-	-	15.0	100.0	-	-	3.0	75.0	-	-	-
Yes	0.0	-	-	-	0.0	-	-	-	1.0	25.0	-	-	-
**Maxillary sinus**	-	-	-	-	-	-	-	-	-	-	-	-	0.0290
No	29.0	100.0	-	-	13.0	87.0	-	-	2.0	50.0	-	-	-
Yes	0.0	-	-	-	2.0	13.0	-	-	2.0	50.0	-	-	-
Total sinuses	-	-	0.0	-	-	-	1.0	0–1	-	-	1.5	0–3.5	0.0001
**Largest tumour dimension (mm)**	-	-	29.5	24.25–34.5	-	-	36.2	24–49	-	-	58.0	45–84	0.0092

HIV, human immunodeficiency virus; IQR, interquartile range.

Owing to the aggressive nature of orbital SCC, surrounding bones tend to be eroded by the tumour. The medial and lateral orbital walls were found to be the most eroded bones. The distribution varied between the different bony walls of the orbit and skull bones, and to a lesser extent with the clivus, orbital fissure and inferior wall. A small subset included the maxillary floor, the optic canal, temporal bone, pterygopalatine fossa, and crista galli (Figure 3). These findings highlight the advanced stage and local aggressiveness of tumours in this series with multi-compartment bony invasion in some individuals. Wang et al. did a retrospective study on 84 patients with lacrimal sac SCC and found in their univariate analysis that orbital bone erosion was one of the poor prognostic factors for 5-year overall survival (94.9% vs 70.2%, *p* < 0.01%). In their multivariate Cox regression analysis, they demonstrated that orbital bone erosion was a poor predictor for progression free survival (87.9% vs 62.9% *p* = 0.023).^26^ Our study did not look at long term disease outcomes.

**FIGURE 3:**
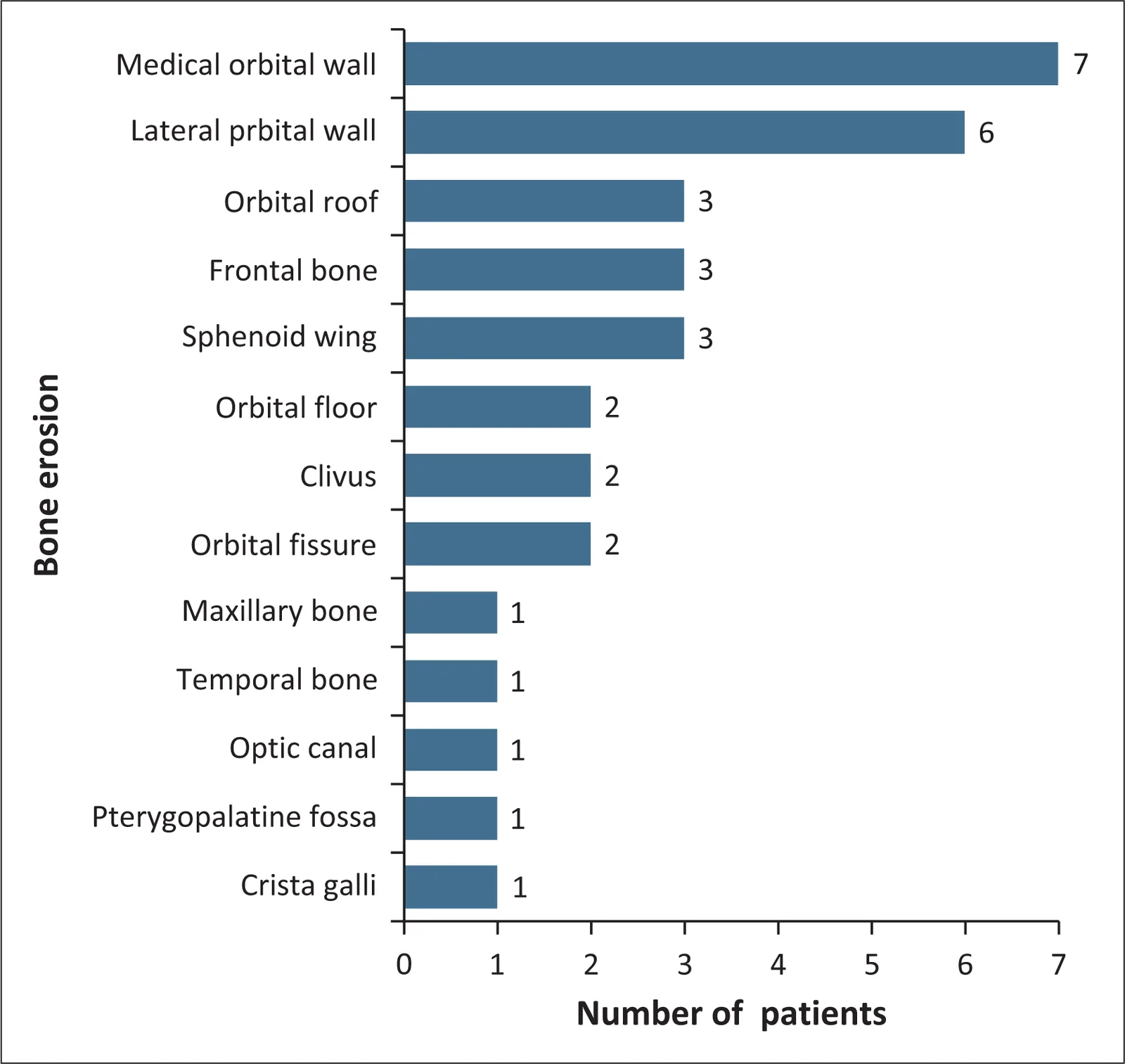
Horizontal bar graph showing radiological bone erosion.

A study by Sakai et al. reported that metastasis to the skull base commonly affects the clivus, apex of the petrosal bone and sphenoid bone. They attributed these findings to the abundance of bone marrow in these bones and that haematogenous spread was the commonest route of metastasis.^27^ A small subset of our study patients had metastatic spread (*p* = 0.001) however because of our sample size, these data may not entirely be representative of metastatic spread in advanced orbital SCC (Table 3).

In our study, the inferior rectus muscle was frequently involved with 58.8% of the patients, followed by medial rectus muscle (Figure 4). However, there is limited published data describing patterns of extraocular muscle involvement in orbital SCC, making it difficult to determine if this distribution is across other populations. Further studies with radiologic and intraoperative correlation may better characterise pattern of extraocular muscle involvement and their clinical significance.

**FIGURE 4:**
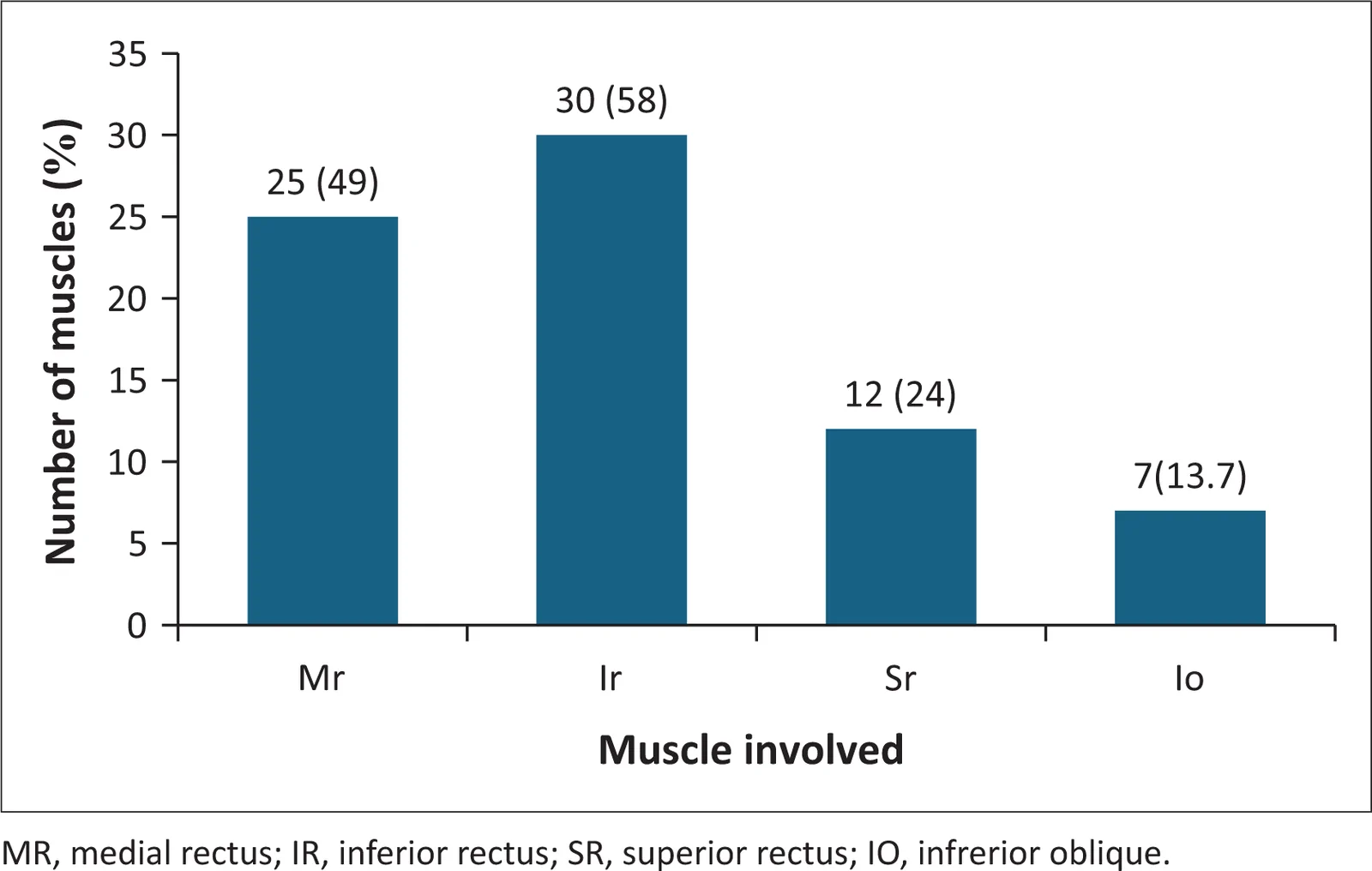
Vertical bar graph showing radiological muscle involvement.

According to the fifth South African National HIV prevalence, incidence, behaviour and communication survey of 2017 (SABSSM V^1^) the Gauteng population between ages 15 years and 64 years had an HIV prevalence of 20.0% (18.7–21.4) at a 95% CI.^28^ Our study found that 82% of patients were HIV positive (Table 1), most of these patients were younger and African suggesting a strong correlation between SCC and HIV disease and the likelihood of conjunctival origin if not elucidated by the patient’s history. This is supported in literature by the fact that HIV disease is one of the important risk factors for SCC because of its association with HPV infections.^3^ A remarkable proportion of the patients had extensive globe involvement, occurring in 90% of the patients (Table 1).

Radiologically we found that there were patients with paranasal sinus involvement, predominantly the ethmoid sinus being affected in 20% of the patients (Table 1). This result was consistent with the finding that the ethmoid sinus was the commonly affected sinus in the spread of orbital SCC.^8,9^ Because of the orbit’s adjacency to the skull base and paranasal sinuses, tumours arising from one structure can spread to the other.^29^

Our study looked at advanced disease, therefore there is no indication that the tumours with paranasal sinus involvement originated from there. A small subset of patients had variable involvement across one or more sinuses (Table 3). There is no clearly documented management approach in literature as to how advanced orbital SCC is managed. Patients can be treated with surgery, chemotherapy, radiation therapy or immune checkpoint inhibitors.^20^ Orbital exenteration is opted for in locally aggressive and advancing disease. In the case series by Rana et al.,^24^ although their patients were treated with immunotherapy, 2 of the 10 patients eventually required orbital exenteration for progressive and residual disease. Our patients received orbital exenteration as a form of treatment due to local invasion of the orbit by the tumour. A high rate (56.7%) of orbital exenteration in the management of SCC in Zimbabwe was attributed to patients presenting with advanced disease, aggressive type of tumour and link with HIV infection.^30^ Our choice of treatment was governed by institutional protocols and resource availability.

### Strengths and limitations

This is the first study with a large cohort of patients compared to what has been documented in the literature. Owing to the few documented studies such as this one in the literature, comparative data with the rest of the world could not be extrapolated. Small sample size made it challenging to make inferences about certain findings. The paucity of data in the literature regarding radiological characteristics of orbital SCC and extensive intra-operative findings makes it challenging to make correlations between parameters.

## Conclusion

Orbital SCC requiring exenteration has a varied radiological presentation. A large proportion of the patients are HIV positive, much higher than the population prevalence suggesting a strong correlation. Patients with disease confined to the orbit did not have lymph node involvement, bone erosion, intracranial spread, distant metastases or sinus involvement. Optic nerve involvement was associated with more aggressive features and a greater propensity to spread beyond the orbit. Conjunctival SCC is an AIDS defining entity and because most patients are HIV positive, would suggest that this was the main cause of the orbital disease through delayed attendance or management.
